# Genetic Mapping of the *HLA1* Locus Causing Hybrid Lethality in *Nicotiana* Interspecific Hybrids

**DOI:** 10.3390/plants10102062

**Published:** 2021-09-30

**Authors:** Takahiro Tezuka, Naoto Kitamura, Sae Imagawa, Akira Hasegawa, Kumpei Shiragaki, Hai He, Masanori Yanase, Yoshiyuki Ogata, Toshinobu Morikawa, Shuji Yokoi

**Affiliations:** 1 Graduate School of Life and Environmental Sciences, Osaka Prefecture University, Osaka 599-8531, Japan; naoto-kitamura@takii.co.jp (N.K.); shiragakikumpei@hotmail.co.jp (K.S.); haihe2020@hotmail.com (H.H.); myanase@plant.osakafu-u.ac.jp (M.Y.); ogata@plant.osakafu-u.ac.jp (Y.O.); d-morikawa@hannan-u.ac.jp (T.M.); shyokoi@plant.osakafu-u.ac.jp (S.Y.); 2 Education and Research Field, College of Life, Environment and Advanced Sciences, Osaka Prefecture University, Osaka 599-8531, Japan; 3 School of Life and Environmental Sciences, Osaka Prefecture University, Osaka 599-8531, Japan; hd2s.24t-gk.ns@softbank.ne.jp (S.I.); chushin-no-nishi-nokai@pref.kyoto.lg.jp (A.H.); 4 Bioeconomy Research Institute, Research Center for the 21st Century, Osaka Prefecture University, Osaka 599-8531, Japan

**Keywords:** hybrid lethality, interspecific population, linkage analysis, *Nicotiana debneyi*, *Nicotiana fragrans*, reproductive isolation

## Abstract

Hybrid lethality, a postzygotic mechanism of reproductive isolation, is a phenomenon that causes the death of F_1_ hybrid seedlings. Hybrid lethality is generally caused by the epistatic interaction of two or more loci. In the genus *Nicotiana*, *N. debneyi* has the dominant allele *Hla1-1* at the *HLA1* locus that causes hybrid lethality in F_1_ hybrid seedlings by interaction with *N. tabacum* allele(s). Here, we mapped the *HLA1* locus using the F_2_ population segregating for the *Hla1-1* allele derived from the interspecific cross between *N. debneyi* and *N. fragrans*. To map *HLA1*, several DNA markers including random amplified polymorphic DNA, amplified fragment length polymorphism, and simple sequence repeat markers, were used. Additionally, DNA markers were developed based on disease resistance gene homologs identified from the genome sequence of *N. benthamiana*. Linkage analysis revealed that *HLA1* was located between two cleaved amplified polymorphic sequence markers Nb14-CAPS and NbRGH1-CAPS at a distance of 10.8 and 10.9 cM, respectively. The distance between these markers was equivalent to a 682 kb interval in the genome sequence of *N. benthamiana*.

## 1. Introduction

Species are often reproductively isolated from each other. This mechanism, called reproductive isolation, has been an interesting theme in evolutionary biology because it plays an important role in preventing gene flow between species [1]. However, reproductive isolation is an obstacle that should be resolved for plant breeding. Although many studies have reported the prevention or bypass of reproductive isolation [2,3,4], challenging cases are still often encountered.

Developmental defects of seedlings such as hybrid lethality, hybrid necrosis, and hybrid weakness are often observed in F_1_ [5,6,7] or later generations (hybrid breakdown) [8,9] in many plant species. These phenomena are caused by epistatic interactions of two or more loci as explained by the Bateson–Dobzhansky–Muller model [10]. Many of the identified causal genes for seedling developmental defects encode disease resistance proteins [11,12,13,14]. In other cases, seedling developmental defects were also reported to be caused by duplicate genes such as *histidinol-phosphate amino-transferase* gene [15] or photosynthetic gene *plastid transcriptionally active chromosome 14* [16].

In the genus *Nicotiana*, hybrid lethality that causes death of F_1_ hybrid seedlings is often observed in interspecific crosses including those involving cultivated species *N. tabacum* [17,18]. Many types of hybrid lethality are temperature sensitive and suppressed at elevated temperatures ranging from 32 °C to 36 °C [19,20,21]. Several mechanisms which explain how hybrid lethality occurs have been revealed: programmed cell death with apoptotic features [22,23], generation of reactive oxygen species [24], and ethylene [25], and autophagy [26] are observed during hybrid lethality. Considering that some types of disease resistance responses are temperature sensitive [27,28,29] and involve programmed cell death [30], the results from the experiments on hybrid lethality in *Nicotiana* have suggested that disease resistance-related responses might be associated with hybrid lethality. Several genes and proteins also relevant to disease resistance have been identified in the process of hybrid lethality [31,32,33,34]. However, the causal genes for hybrid lethality in *Nicotiana* are poorly understood.

Hybrid lethality in *Nicotiana* is thought to be caused by epistatic interactions between alleles at different loci [18,35]. Previous studies have demonstrated that the Q chromosome of the S genome in *N. tabacum* has gene(s) that cause hybrid lethality [17,20,36,37]. Recently, the *N. tabacum hybrid lethality 1* (*NtHL1*) gene encoding a protein containing a coiled-coil, nucleotide-binding site, and leucine-rich repeat was identified in *N. tabacum* [38]; this gene might correspond to the hypothesized gene(s) of the epistatic interaction on the Q chromosome. For the counterpart of the Q chromosome gene(s), we identified the *hybrid lethality A1* (*HLA1*) gene that causes hybrid lethality by interaction with the hypothesized gene(s) on the Q chromosome [39]. Wild species in *Nicotiana* section *Suaveolentes*, *N. debneyi*, has the dominant allele *Hla1-1* at the *HLA1* locus; we obtained F_1_ hybrids between *N. debneyi* and *N. fragrans* in which the latter also belongs to section *Suaveolentes* and possesses the non-causal allele *hla1-2* at the *HLA1* locus. The F_1_ hybrids were self-fertile, and F_2_ seeds were obtained [39]. Therefore, the *HLA1* locus can be mapped to a specific region of a chromosome. However, the chance of crossover depends on whether chromosome pairing occurs. In interspecific hybrids, chromosome pairing is often disturbed due to differences in the genomes or chromosomes of both parents [40,41].

In the present study, we aimed to map the position of the *HLA1* locus. Linkage analysis was carried out using the F_2_ population derived from the cross *N. debneyi* × *N. fragrans* with several DNA markers. Because little genomic or marker information was available for the parental species *N. debneyi* and *N. fragrans* (both allotetraploids), we initially used random amplified polymorphic DNA (RAPD) and amplified fragment length polymorphism (AFLP) markers [42,43] that do not require previous sequence information, as well as simple sequence repeat (SSR) markers developed in *N. tabacum* [44]. In many cases, disease resistance-related genes have been identified as causal genes for hybrid lethality [11,12,13,14,38]. Therefore, it is reasonable to assume that *HLA1* might be a disease resistance-related gene. One parent, *N. debneyi*, shows several disease resistances, some of which have been transferred to *N. tabacum* [45,46,47]. We used two reported sequence-characterized amplified region (SCAR) markers, SUBC180.251 [48] and Mil275 [49], linked to blue mold resistance in *N. tabacum* that was originally transferred from *N. debneyi*. To develop further DNA markers, we also used the draft genome sequence of *N. benthamiana* [50], a species belonging to the section *Suaveolentes* and closely related to *N. debneyi* and *N. fragrans* [51,52]. Additionally, we investigated chromosome pairing in the F_1_ hybrid between *N. debneyi* and *N. fragrans*. Here, we report the map position of the *HLA1* locus in the linkage map derived from the F_2_ population of the cross *N. debneyi* × *N. fragrans*.

## 2. Results

### 2.1. Segregation of the Hybrid Lethality Phenotype in the F_2_ Population

After the test crosses with *N. tabacum*, crossed seeds were obtained from 81 of 125 F_2_ plants derived from the cross *N. debneyi* × *N. fragrans*; seeds could not be obtained in crosses using the remaining 44 F_2_ plants. The F_2_ plants segregated into plants yielding only inviable progeny:plants yielding segregating progeny:plants yielding only viable progeny = 21:45:15, fitting well with the expected 1:2:1 ratio (χ^2^ = 1.89, *p* > 0.05) in F_2_ plants. This was consistent with a previous study showing that *N. debneyi* has a single dominant gene (*Hla1-1*) causing hybrid lethality [39].

### 2.2. Construction of a Linkage Map Using RAPD, AFLP, and SSR Markers

**Figure 1:**
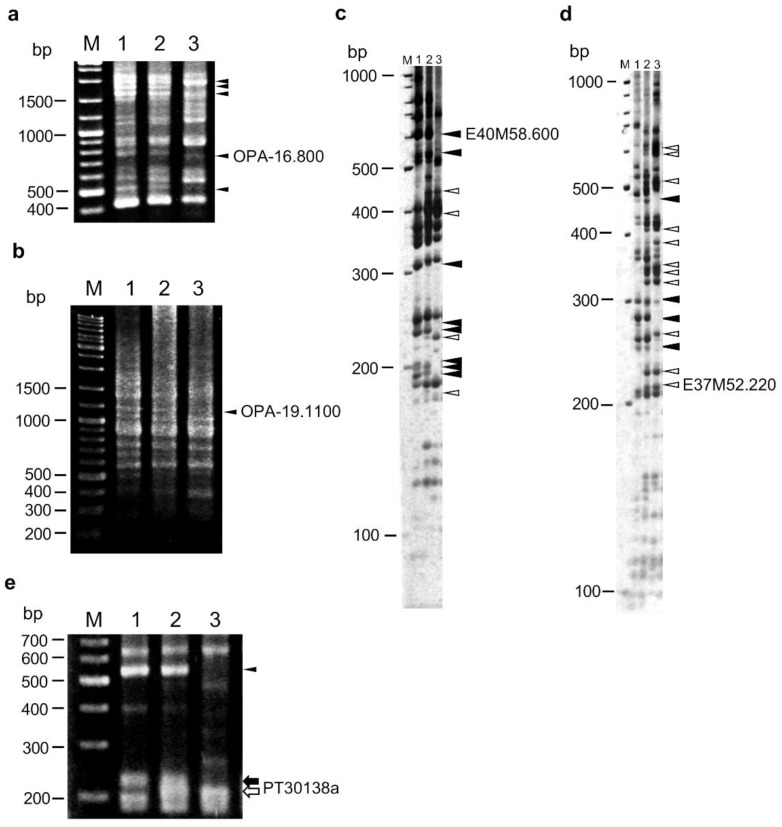
RAPD, AFLP, and SSR markers linked to the *HLA1* locus. (**a**,**b**) RAPD patterns amplified with primers OPA-16 (**a**) and OPA-19 (**b**). (**c**,**d**) AFLP patterns amplified with primers E40 and M58 (**c**), and primers E37 and M52 (**d**). (**e**) SSR pattern amplified with primers for PT30138 marker. Lanes: M, size marker (GeneRuler DNA ladder mix; Thermo Fisher Scientific, Tokyo, Japan); 1, *N. debneyi*; 2, F_1_ hybrid between *N. debneyi* and *N. fragrans*; 3, *N. fragrans*. Black and white arrowheads indicate dominant markers specific to *N. debneyi* and *N. fragrans*, respectively. Black and white arrows indicate codominant marker PT30138a bands specific to *N. debneyi* and *N. fragrans*, respectively.

RAPD, AFLP, and SSR markers were initially used to map the *HLA1* locus. In RAPD analysis, 385 bands were detected using 80 random primers; 78 (20.3%) bands showed polymorphism between *N. debneyi* and *N. fragrans* (Figure 1a,b; Table 1). In AFLP analysis, 2327 (44.8%) of 5191 bands detected using 256 primer pairs showed polymorphism between the parents (Figure 1c,d; Table 1). These polymorphic bands were assumed to be dominant markers.

For SSR analysis, 112 bands were detected using 66 primer pairs; 22 and 12 bands were specific to *N. debneyi* and *N. fragrans*, respectively, and 14 bands were codominant. Therefore, although SSR markers were generally codominant, only seven markers were detected as codominant in the present study. Totally, 41 markers were detected as polymorphic (Figure 1e, Table 1).

Of the above-identified polymorphic markers, all 78 RAPD markers, 111 AFLP markers, and 13 SSR markers were used to construct a linkage map. Linkage analysis resulted in only two major (LG1 and LG2) and 24 minor linkage groups (LG3 to LG26). Within the minor linkage groups, map distances of LG3 to LG8 were comparatively longer than others (Figure S1). Many markers tended to cluster in some regions. The *HLA1* locus was mapped between the codominant SSR marker PT30138a and the AFLP marker E40M58.600 in LG1.

### 2.3. Chromosome Pairing in F_1_ Hybrid between N. debneyi and N. fragrans

Because many linkage groups were generated, we investigated chromosome pairing in the F_1_ hybrid used to produce the F_2_ generation to verify whether crossover can occur during meiosis of F_1_ hybrids between *N. debneyi* and *N. fragrans*. A high frequency of bivalents was observed with an average of 0.6 univalents, 23.2 bivalents, and 0.4 trivalents per pollen mother cell (Figure S2; Table S1).

### 2.4. Linkage Analysis of HLA1 Using Disease Resistance-Related Markers

When two SCAR markers linked to blue mold resistance were tested, SUBC180.251 showed no polymorphism between parents, whereas Mil275 was detected in *N. debneyi* and the F_1_ hybrid but not in *N. fragrans* (Figure S3a,c). SUBC180.251 was converted to the cleaved amplified polymorphic sequence (CAPS) marker SUBC180.251-CAPS, and this newly developed marker distinguished the genotypes of both parents and the F_1_ hybrid (Figure S3b). Both SUBC180.251-CAPS and Mil275 were mapped to LG2 and thus were not linked to the *HLA1* locus.

**Figure 2:**
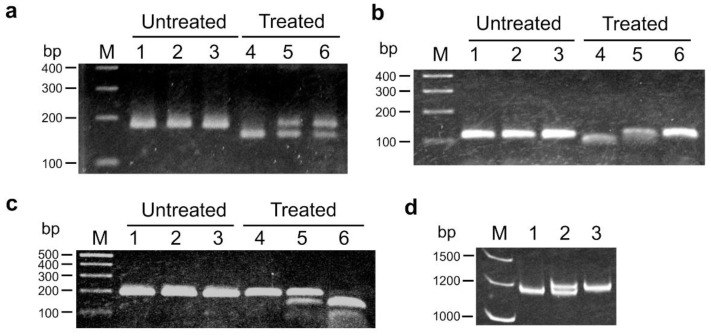
DNA markers developed based on v0.4.4 genome of *N. benthamiana*. (**a**–**c**) CAPS markers NbRGH1-CAPS (a) and NbRGH3-CAPS (c), and dCAPS marker NbRGH2-dCAPS (**b**). Undigested PCR products (lanes 1–3) and PCR products treated with appropriate restriction enzymes (lanes 4–6) were detected by agarose gel electrophoresis. (**d**) STS marker NbRGH4 detected by polyacrylamide gel electrophoresis. Lanes: M, size marker (GeneRuler DNA ladder mix); 1, 4, *N. debneyi*; 2, 5, F_1_ hybrid between *N. debneyi* and *N. fragrans*; 3, 6, *N. fragrans*.

**Figure 3:**
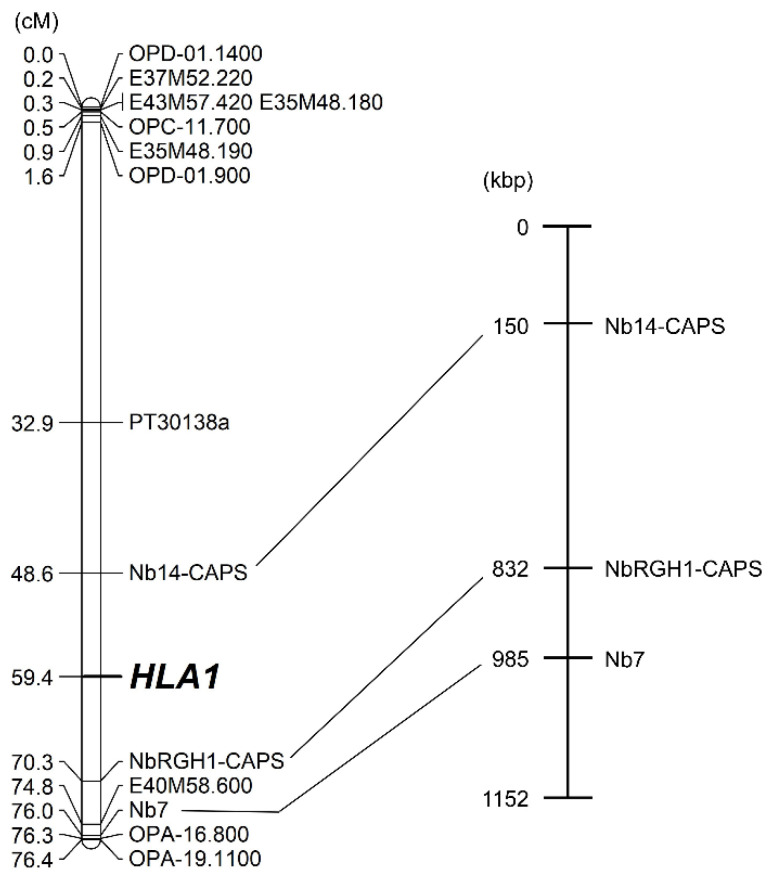
Linkage map (**left**) and physical map (**right**) indicating the position of the *HLA1* locus. The physical map was based on the v1.0.1 genome of *N. benthamiana*.

We then used the draft genome sequence of *N. benthamiana* [50]. When we started the experiments, the *N. benthamiana* genome v0.4.4 was available. To identify resistance gene homologs from the genome sequence, a BLAST search was conducted using TIR-NBS-LRR resistance genes in *Arabidopsis*. Four disease resistance gene homologs were identified from the *N. benthamiana* protein and transcript sequences (Table S2). Four sequence-tagged site (STS) markers, NbRGH1 to NbRGH4, were developed based on the sequences of their homologs (Table S3). Among these markers, only NbRGH4 showed polymorphism in the parents and F_1_ hybrid (Figure 2d). After the conversion of the remaining three markers to CAPS or derived CAPS (dCAPS) markers, NbRGH2-dCAPS and NbRGH3-CAPS could be used as codominant polymorphic markers (Figure 2b,c). For NbRGH1-CAPS, a lower band resulting from *Bsr*I digestion was detected in *N. debneyi* as expected, but two bands were unexpectedly detected in *N. fragrans* (Figure 2a); this might be due to the complexity of the *N. fragrans* genome because this species is an allotetraploid. Linkage analysis revealed that NbRGH1-CAPS was linked to the *HLA1* locus at a distance of 10.9 cM (Figure 3). Two markers were mapped to other linkage groups: NbRGH3-CAPS to LG2 and NbRGH4 to LG3 (Figure S1). No marker linked to NbRGH2-dCAPS was detected.

**Figure 4:**
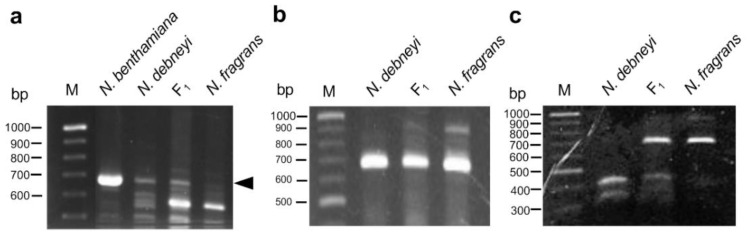
DNA markers developed based on the scaffold Niben101Scf06736 in the v1.0.1 genome of *N. benthamiana*. (**a**) STS marker Nb7 (arrowhead). (**b**,**c**) CAPS marker Nb14-CAPS. Undigested PCR products (**b**) and PCR products treated with *Hha*I (**c**) are shown. Lane M, size marker (GeneRuler DNA ladder mix).

Because NbRGH1-CAPS, involved in the Niben044Scf00009821 in the v0.4.4 scaffold, was linked to the *HLA1* locus, additional markers were developed using the v1.0.1 scaffold released during the conduct of this study. The Niben044Scf00009821 scaffold was involved in the Niben101Scf06736 scaffold in v1.0.1. Based on the scaffold information, we developed two STS markers: Nb7 and Nb14 (Table S3). Nb7 could be used as a dominant polymorphic marker (Figure 4a). Although Nb14 showed no apparent polymorphism between parents in agarose gel electrophoresis, the CAPS marker Nb14-CAPS, developed based on genome sequences of the parents at Nb14, showed polymorphism (Figure 4b,c). Eventually, the *HLA1* locus was located between Nb14-CAPS and NbRGH1-CAPS at a distance of 10.8 and 10.9 cM to *HLA1*, respectively (Figure 3). The distance between these markers was equivalent to a 682-kb interval in the genome sequence of *N. benthamiana*.

## 3. Discussion

In the genus *Nicotiana*, only limited interspecific crosses have been used to produce segregating populations available to construct linkage maps, for example, the cross between *N. plumbaginifolia* and *N. longiflora* [53]. Both *N. debneyi* and *N. fragrans* have 48 chromosomes and are closely related to each other [51,52]. Furthermore, a high frequency of bivalents was observed in the F_1_ hybrid between *N. debneyi* and *N. fragrans*, suggesting that crossover would occur during meiosis. As a result, the *HLA1* locus could be mapped to linkage group LG1 (Figure 3 and Figure S2).

Compared to RAPD analysis, AFLP analysis amplified a greater number of bands per primer (5191/256 in AFLP against 385/80 in RAPD) and yielded a higher percentage of polymorphisms (Table 1). However, both types of DNA markers tended to cluster in some regions. In this regard, clustering of AFLP markers has often been reported [54]. SSR markers that were developed in *N. tabacum* also showed a higher percentage of polymorphisms than RAPD markers (Table 1). Although several SSR markers were dominantly scored; this might be because *N. tabacum* SSR markers amplified only single bands even in *N. tabacum* and showed somewhat low transferability to other *Nicotiana* species: 72% transferability for *N. africana*, 41% for *N. benthamiana*, and 50% for *N. suaveolens* [44,55].

Several markers deviated from expected Mendelian ratios were observed (Figure S1). Segregation distortion might be caused by several reproductive isolation mechanisms [56,57]. Therefore, some reproductive isolation mechanisms might exist between *N. debneyi* and *N. fragrans*.

Because *N. debneyi* and *N. fragrans* possess 48 chromosomes, it is expected that the saturated linkage map consists of 24 linkage groups when constructed using the F_2_ population from the cross *N. debneyi* × *N. fragrans*. In the present study, however, the linkage map consisted of only two major (LG1 and LG2) and 24 minor linkage groups (Figure S1). This might be because of the inadequate number of markers for linkage map construction and/or the clustering of many markers. Nevertheless, considering that markers were not distributed uniformly among all linkage groups, it is more likely that enough crossover events did not occur, although a high frequency of bivalents was observed in the F_1_ hybrid. Therefore, using the F_2_ population from the cross *N. debneyi* × *N. fragrans*, only limited positions of loci such as the *HLA1* locus can be mapped through linkage analysis.

Based on the *N. benthamiana* v1.0.1 genome scaffold, nine genes were found in the region between Nb14-CAPS and NbRGH1-CAPS where the *HLA1* locus was mapped (Table S4). Among these genes, three genes (Scf06736g04011.1, Scf06736g06007.1, and Scf06736g08001.1) might be associated with plant responses to pathogens based on the description available at the Sol Genomics Network. Therefore, these genes may be good candidates for *HLA1*. Nevertheless, we cannot exclude the possibility that another locus gene is responsible for *HLA1* because different genes might exist between *N. benthamiana* and *N. debneyi* or *N. fragrans* in the concerned region due to possible chromosomal differences generated during the process of speciation. Fine mapping is required to definitively identify candidate genes for *HLA1*.

## 4. Materials and Methods

### 4.1. Plant Materials

An F_1_ hybrid between *N. debneyi* (2*n* = 48; homozygous for *Hla1-1*) and *N. fragrans* (2*n* = 48; homozygous for *hla1-2*) obtained in a previous study [39] was used to produce the F_2_ population. A total of 125 F_2_ plants were used as mapping populations. Each F_2_ plant was test-crossed with *N. tabacum* ‘Samsun NN’ (2*n* = 48) to estimate the genotype at the *HLA1* locus. The seeds were aseptically sown on 1/2 MS medium [58] supplemented with 1% sucrose and 0.2% Gelrite (pH 5.8) and then cultured at 25 °C (16 h light/8 h dark, approximately 100 µmol m^−2^ s^−1^). At least 10 plants per each cross were observed for lethality. F_2_ plants were determined to be homozygous for *Hla1-1* when all the progeny obtained from the cross with *N. tabacum* were inviable, heterozygous when the progeny was segregated for viable and inviable (approximately 1:1), and homozygous for *hla1-2* when all the progenies were viable.

### 4.2. DNA Extraction

Total DNA was extracted from the leaves of parental, F_1_, and F_2_ plants using the cetyltrimethylammonium bromide (CTAB) method [59] and used for linkage analysis using DNA markers.

### 4.3. RAPD Analysis

RAPD analysis was carried out as described by Williams et al. [42] with some minor modifications. Briefly, 80 random 10-mer oligonucleotide primers (Kit A to D) were obtained from Operon Technologies (Alameda, CA, USA). Reaction mixtures contained 1× Robust Buffer (BioAcademia, Osaka, Japan), 0.2 mM each dNTP, 0.5 µM primer, 10 ng template DNA, and 0.5 U Taq DNA polymerase (BioAcademia) in a total volume of 10 µL. PCR amplification was performed using a TProfessional Basic Thermocycler (Biometra, Göttingen, Germany) programmed for 1 min at 94 °C for initial denaturation, followed by 45 cycles of 30 s at 94 °C, 1 min at 36 °C, 2 min at 72 °C, and a final extension of 3 min at 72 °C. PCR products were separated by electrophoresis in a 1.5% agarose gel in TBE buffer and stained with ethidium bromide to visualize the DNA bands. During the analysis, only intense and clear DNA bands were observed. Each RAPD marker was named as the primer and the subsequent number that indicated the estimated DNA fragment size.

### 4.4. AFLP Analysis

AFLP analysis was carried out as described by Vos et al. [43] with some modifications. Total DNA was double-digested with *Eco*RI and *Mse*I and ligated to the *EcoRI adaptor* and the *MseI adaptor*. The preamplification reaction was carried out using *Eco*RI+A (E01) and *Mse*I+C (M02) primers (Table S5). After the reaction, the PCR product was diluted 10-fold with sterilized distilled water and used as a template for the second amplification reaction. Primers with three selective nucleotides, 16 *Eco*RI+ANN (N indicates A, T, G, or C; E31 to E46) and 16 *Mse*I+CNN (M47 to M62) primers (Table S5), were used for the second amplification reaction with all possible combinations (256 primer pairs). In both amplification reactions, KAPA Taq EXtra DNA polymerase (Kapa Biosystems, Wilmington, MA, USA) was used as the enzyme. PCR amplification was performed using the PC-818A Program Temp Control System (Astec, Fukuoka, Japan). The second PCR products were separated by electrophoresis on an 8% polyacrylamide gel and silver stained [60]. Each AFLP marker was named as the primer combination and subsequent number that indicated the estimated DNA fragment size.

### 4.5. SSR Analysis

An *N. tabacum* linkage map consisting of 24 linkage groups was constructed using SSR markers in *N. tabacum* [44]. Sixty-six SSR markers (Table S6) were selected to cover all linkage groups in *N. tabacum* and were detected as follows: Reaction mixtures contained 1× Robust Buffer (BioAcademia), 0.2 mM each dNTP, 0.2 µM of each primer, 10 ng template DNA, and 0.25 U Taq DNA polymerase (BioAcademia) in a total volume of 10 µL. PCR amplification was performed using a TProfessional Basic Thermocycler (Biometra) programmed for 3 min at 94 °C for initial denaturation, followed by 35 cycles of 30 s at 94 °C, 1 min at 50 °C, 5 s at 72 °C, and a final extension of 5 min at 72 °C. PCR products were separated by electrophoresis in a 3% agarose gel in TBE buffer and stained with ethidium bromide to visualize the DNA bands. If several loci were detected using one primer pair, they were distinguished by adding a lower-case letter to the end of the marker name.

### 4.6. DNA Markers Related to Disease Resistance

Two SCAR markers—SUBC180.251 [48] and Mil275 [49]—linked to blue-mold resistance in *N. tabacum* were used, assumed to be introduced from *N. debneyi* [48,49]. These markers were detected using conventional PCR.

The draft genome sequence of *N. benthamiana* [50] was released through the Solanaceae (Sol) Genomics Network [61] (https://solgenomics.net/, accessed on 27 September 2021). Because disease resistance-related genes were not identified based on the genome sequence at the time of initiation of this research, we searched for disease resistance gene homologs. One-hundred-and-four TIR-NBS-LRR proteins in *Arabidopsis thaliana* were identified based on the protein sequences (TAIR10_pep_20101214) obtained from The Arabidopsis Information Resource [62] (https://www.arabidopsis.org/, accessed on 27 September 2021) and were used as query sequences in BLASTP searches (*e*-value < 1 × 10^−5^) against the *N. benthamiana* protein sequences (Niben.genome.v0.4.4.proteins.annotated.fasta), downloaded from the Sol Genomics Network. Based on the *N. benthamiana* proteins identified in the searches, the corresponding transcripts were identified from *N. benthamiana* transcript sequences (Niben.genome.v0.4.4.transcripts.annotated.fasta) downloaded from the Sol Genomics Network. To determine the genomic regions encoding the transcripts identified, the transcript sequences were used as query sequences in BLASTN searches against the *N. benthamiana* genome scaffolds (Niben.genome.v0.4.4.scaffolds.nrcontigs.fasta) downloaded from the Sol Genomics Network. Based on the genomic sequences of four disease resistance gene homologs identified in *N. benthamiana* (Table S2), four STS markers, NbRGH1 to NbRGH4, were developed (Table S3). Subsequently, two STS markers, Nb7 and Nb14, located on the same scaffold as NbRGH1, were developed based on the newly available *N. benthamiana* genome scaffolds (Niben.genome.v1.0.1.scaffolds.nrcontigs.fasta) from the Sol Genomics Network. Primers for each marker were designed using Primer3 software [63]. After conventional PCR amplification, the PCR products were separated by electrophoresis on a 1.5% agarose gel or 8% polyacrylamide gel (only NbRGH4).

For five markers (SUBC180.251, NbRGH1, NbRGH2, NbRGH3, and Nb14), the DNA fragments corresponding to each marker were completely (SUBC180.251 and Nb14) or partly (NbRGH1, NbRGH2, and NbRGH3) directly sequenced from both parents, and converted to CAPS or dCAPS markers (Table S3). For four markers (NbRGH1-CAPS, NbRGH2-dCAPS, NbRGH3-CAPS, and Nb14-CAPS), primers were newly designed using Primer3 software. We also used dCAPS Finder 2.0 [64] to design primers for the dCAPS marker NbRGH2-dCAPS. After digestion of the PCR products with the corresponding restriction enzymes, the products were separated by electrophoresis on a 3.0% agarose gel.

### 4.7. Linkage Analysis

After determining the *HLA1* genotype and scoring the genotypes at DNA markers in F_2_ plants, each locus was tested for goodness of fit to the expected 3:1 (dominant) or 1:2:1 (codominant) ratios by a chi-square test at the 5% level of significance. The linkage map was constructed using JoinMap 4.1 [65] with an LOD of 4.0. Recombination values were converted to map distances using the Kosambi mapping function.

### 4.8. Cytological Analysis of Chromosome Pairing

Chromosome pairing in pollen mother cells during meiotic metaphase I was investigated to examine whether recombination was expected to occur in the F_1_ hybrid used to produce the F_2_ population. Anthers collected from young flower buds were fixed in chloroform/ethanol/acetic acid (6:3:1). The anthers were then placed on a glass slide and squashed in acetocarmine. Chromosome pairing was observed in 25 pollen mother cells from the F_1_ hybrid using a light microscope (Optiphot-2; Nikon, Tokyo, Japan).

## Figures and Tables

**Table 1:** Polymorphic markers detected by RAPD, AFLP, and SSR analyses.

Marker Type	No. of Primers	No. of Bands Detected				Percentage of Polymorphic Bands	Total No. of Polymorphic Markers
		Total	*N. debneyi*-Specific Dominant	*N. fragrans*-Specific Dominant	Codominant		
RAPD	80	385	42	36	0	20.3%	78
AFLP	256 sets	5191	1176	1151	0	44.8%	2327
SSR	66 sets	112	22	12	14 (7 sets)	42.9%	41

## Data Availability

The data presented in this study are available in article and supplementary material.

## Supplementary Materials

The following are available online at https://www.mdpi.com/article/10.3390/plants10102062/s1, Figure S1: Linkage map derived from the F_2_ population from the cross *N. debneyi* × *N. fragrans*, Figure S2: Chromosome pairing in a pollen mother cell of an F_1_ hybrid from the cross *N. debneyi* × *N. fragrans*, Figure S3: DNA markers linked to blue-mold resistance, Table S1: Chromosome pairing in an F_1_ hybrid between *N. debneyi* and *N. fragrans*, Table S2: TIR-NBS-LRR resistance gene homologues identified in *N. benthamiana* through BLAST search, Table S3: DNA markers developed for *HLA1* mapping, Table S4: Genes between Nb14-CAPS and NbRGH1-CAPS markers in *N. benthamiana* v1.0.1 genome, Table S5: Primers used for AFLP analysis, Table S6: SSR markers used in *HLA1* mapping.
