# Supplementary material for: Genetic Mapping of the HLA1 Locus Causing Hybrid Lethality in Nicotiana Interspecific Hybrids
Source: Plants (Basel). 2021 Sep 30;10(10):2062. doi: 10.3390/plants10102062 (PMC8539413; doi:10.3390/plants10102062)
Supplement: Supplementary file 1 [file plants-10-02062-s001.zip › Supplementary Figures.pdf]

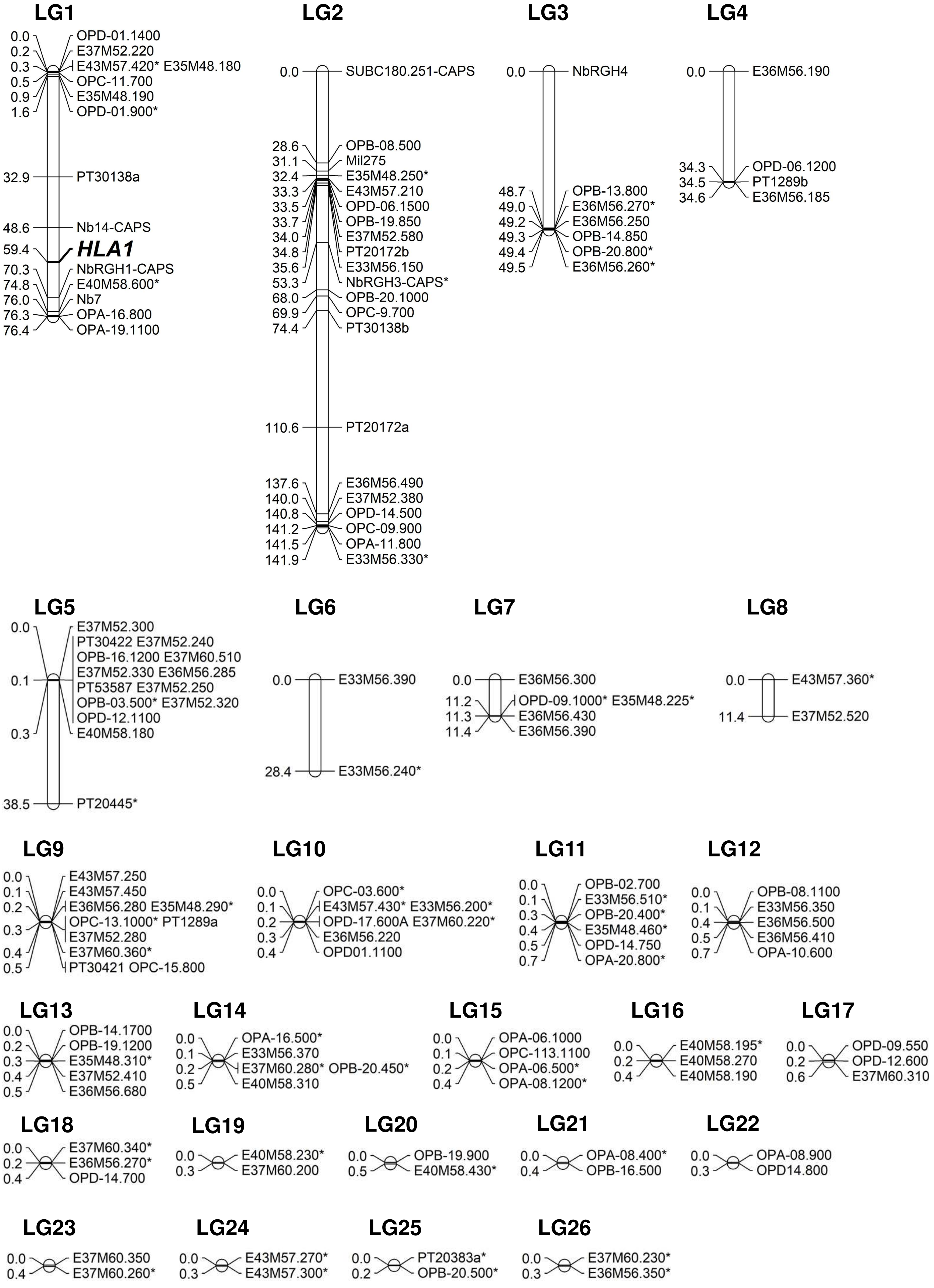

Figure S1. Linkage map derived from the F_2 population from the cross *N. debneyi* × *N. fragrans*. The distance is in cM. DNA markers with an asterisk are those that deviated from Mendelian ratios.

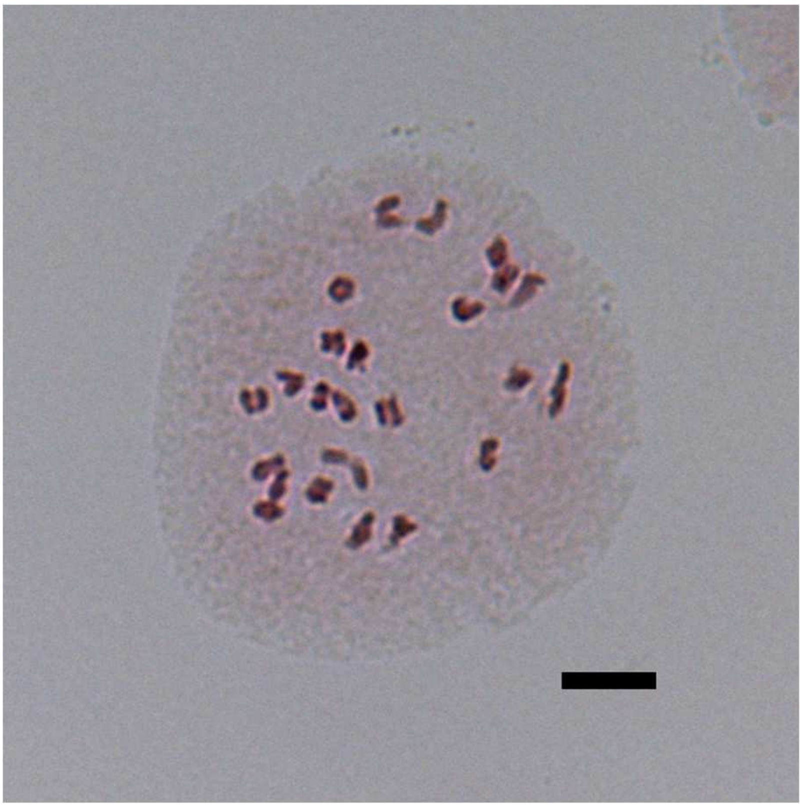

Figure S2. Chromosome pairing in a pollen mother cell of an F_1 hybrid from the cross *N. debneyi* × *N. fragrans*. Twenty-four bivalents were observed at metaphase I of meiosis. Scale bar indicates 10 μm.

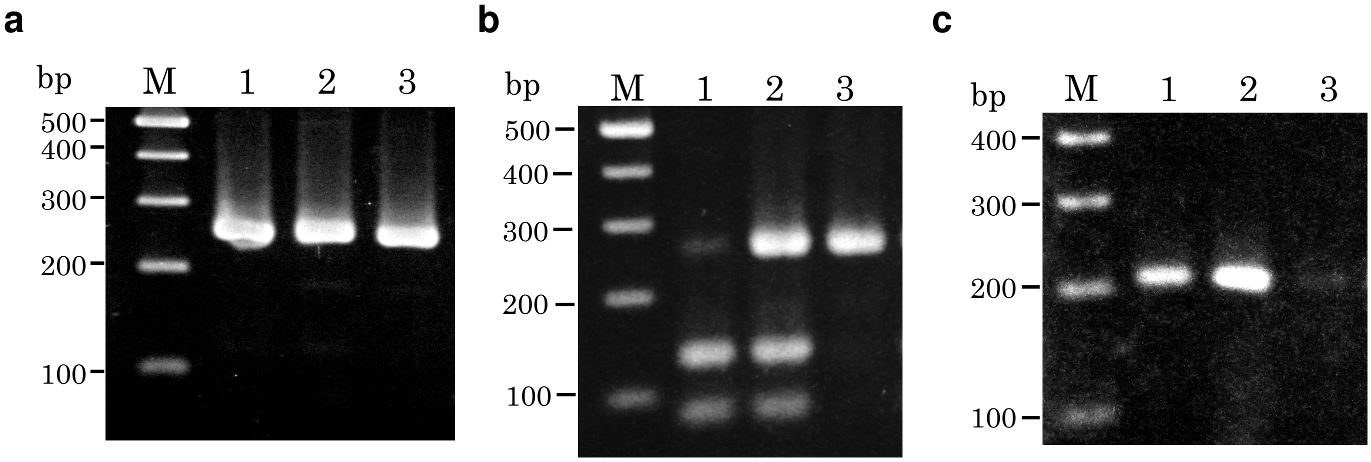

Figure S3. DNA markers linked to blue-mold resistance. **a** SCAR marker SUBC180.251. **b** CAPS marker SUBC180.251-CAPS. **c** SCAR marker Mil275. Lanes: M, size marker (GeneRuler DNA ladder mix; Thermo Fisher Scientific, Tokyo, Japan); 1, *N. debneyi*; 2, F_1 hybrid between *N. debneyi* and *N. fragrans*; 3, *N. fragrans*.
